# Increased soluble phagocytic receptors sMer, sTyro3 and sAxl and reduced phagocytosis in Juvenile-onset Systemic Lupus Erythematosus

**DOI:** 10.1186/s12969-015-0007-y

**Published:** 2015-04-10

**Authors:** Lucy Ballantine, Angela Midgley, David Harris, Ella Richards, Sarah Burgess, Michael W Beresford

**Affiliations:** Department of Women’s and Children’s Health, Institute of Translational Medicine, University of Liverpool, Alder Hey Children’s NHS Foundation Trust Hospital, Eaton Road, Liverpool, L12 2AP UK

**Keywords:** Phagocytosis, TAM receptor, Juvenile-onset SLE, apoptotic cell clearance

## Abstract

**Background:**

The TAM-receptor tyrosine kinase family, Tyro3, Axl and Mer are key to apoptotic cell clearance. Reduced phagocytic clearance in systemic lupus erythematosus (SLE) leads to prolonged exposure of nuclear autoantigen to the immune system. Here we measure the levels of TAM receptors and the phagocytic capacity of monocytes and macrophages in juvenile-onset SLE (JSLE).

**Method:**

Mer protein was measured on monocytes from JSLE, healthy control and JIA patients. JSLE, healthy control and JIA patients’ plasma were analysed for soluble Mer (sMer), soluble Tyro3 (sTyro) and soluble Axl (sAxl). A phagocytosis assay measured the effect of JSLE serum on phagocytic potential of JSLE and control monocytes to engulf *E. Coli* bacteria and healthy macrophages to engulf apoptotic neutrophils.

**Results:**

Mer receptor expression was significantly decreased on JSLE monocytes compared to healthy controls. Plasma sMer, sTyro and sAxl were significantly increased in JSLE patients compared to controls (p < 0.05). Adult healthy control macrophages had significantly decreased phagocytosis of *E. Coli* and apoptotic neutrophils in the presence of 10% JSLE serum compared to control serum (p < 0.05).

**Conclusion:**

JSLE patients have a decreased phagocytosis due to both serum and cell-derived factors. Significantly increased levels of sMer, sTyro3 and sAxl may be important factors contributing to the deficit in phagocytosis ability.

## Background

Phagocytosis is the engulfment and internalisation by phagocytes of particulate targets including microorganisms, apoptotic cells and environmental debris. Characteristically, phagocytic removal of apoptotic cells is a non-inflammatory process, and does not induce an immune response. Juvenile-onset SLE (JSLE) (diagnosed before the age of 17) is generally a more severe disease associated with greater organ damage compared to adult-onset SLE [1]. The etiology of JSLE remains uncertain [2] but is serologically characterised by autoantibodies directed against nuclear autoantigens [3]. It is hypothesised that impaired phagocytosis results in prolonged exposure of apoptotic cells to the immune system leading to production of autoantibodies directed against intracellular nuclear components. Our group has previously demonstrated significantly greater, dysregulated neutrophil apoptosis in JSLE patients compared to healthy controls [4] which have increased surface expression of nuclear antigens [5].

Monocytes and macrophages, two important phagocytes, use surface receptors to perform phagocytic uptake of apoptotic cells. The TAM-receptor tyrosine kinase family consists of three phagocytic receptors: Tyro3, Axl and Mer [6] and are key to apoptotic cell clearance [7] but not required for phagocytosis of bacteria [8]. They recognise the apoptotic cell surface membrane marker phosphatidylserine through two bridging molecules, protein S (ProS) and “a product of growth arrest-specific gene 6” (Gas6).

Mer can also act in an anti-inflammatory manner being primarily expressed on anti-inflammatory M2c macrophages [9]. TAM receptors broadly act through inhibition of Toll-like receptor (TLR) and TLR-induced cytokine-receptor cascades [10]. Gas6 inhibits Tumor Necrosis Factor alpha (TNFα) and Interleukin 6 (IL-6) secretion by Lipopolysaccharide (LPS) stimulated monocytes and macrophages through Mer activation [11]. Cleavage of the extra-cellular membrane bound Mer to form soluble Mer (sMer) occurs via the metalloproteinase A disintegrin and metalloproteinase protein 17 (ADAM17) also known as TACE (tumour necrosis factor-α-converting enzyme) [12]. Mer cleavage leads to receptor inactivation reducing the number of membrane-bound receptors. sMer inhibits macrophage phagocytosis by acting as a decoy-receptor [13] and preventing the anti-inflammatory properties of Gas6-mediated stimulation of membrane-bound Mer. Stimulating monocytes or macrophages with LPS and phorbol-12-myristate-13-acetate (PMA) induces Mer cleavage [13]. Intrinsic monocyte and macrophage phagocyte dysfunction [14] and lupus serum itself may both contribute to impaired phagocytosis in adult onset SLE [15,16]. The aim of this study was to investigate the role of Mer and the three soluble phagocytosis receptors in the phagocytosis potential of monocytes and macrophages in JSLE.

## Methods

### Patients and controls

This study was part of the UK JSLE Cohort Study [17] and approved by the Liverpool Paediatric Research Ethics Committee. Written informed assent/consent was obtained from all participating patients/parents. All JSLE patients fulfilled the revised American College of Rheumatology (ACR) criteria for SLE [18] before the age of 17 years. Paediatric non-inflammatory controls were children without inter-current infection or inflammatory musculoskeletal conditions undergoing elective surgery. All juvenile idiopathic arthritis (JIA) patients met International League of Arthritis and Rheumatism (ILAR) classification criteria [19], serving as an autoimmune inflammatory control. All patients were recruited from outpatient clinics and inpatient wards at Alder Hey Children’s National Health Service (NHS) Foundation Trust. All study samples were anonymized following collection.

### Monocyte separation and macrophage preparation

Immediately following phlebotomy, samples were transferred directly to the laboratory for processing. For monocytes preparation, peripheral blood mononuclear cells (PBMCs) were isolated from fresh heparinised blood within 1 hour of collection by 1-step centrifugation through Polymorph Prep (Axis-shield, Kimbolton, UK) following manufacturer’s instructions. Contaminating erythrocytes were removed by hypertonic lysis. CD14^+^ cells were separated from PBMCs by magnetic selection using CD14^+^ microbeads (Miltenyi Biotech) according to the manufacturer’s instructions. The purity of these cells were assessed and was consistently >95%. For macrophage preparation, the monocytes were differentiated by culturing with 50 ng/ml Macrophage Colony Stimulating Factor (M-CSF) (Miltenyi Biotech) in RPMI-1640 media containing 10% fetal calf serum (FCS) and 1% penicillin/streptomycin (all from Sigma-Aldrich, Poole, UK) for 6 days at 37°C in an atmosphere containing 5% CO_2_. After this duration macrophages were removed from culture plates using cell dissociation solution (Sigma). The macrophages were washed in phosphate buffered saline (PBS; Sigma) and counted before being used for RNA extraction or the phagocytosis assay.

### Phagocytosis assay

A pH sensitive dye pHrodo (Invitrogen) that obtains a red fluorescence under exposure to acidic pH was used for analysing phagocytosis. Phagolysosomes bear an acidic pH, therefore material fully phagocytosed can be quantified.

Monocytes/macrophages from adult healthy control or patients were isolated by the methods outlined and washed twice in Hanks Balanced Salt Solution (HBSS; GIBCO pH 7.4). For the phagocytosis assay, the monocytes/macrophages were incubated with E. Coli prestained with pHrodo in the presence of 10% JSLE, JIA or control serum for 30 minutes at 37°C. As a negative control an identical tube of cells was incubated at 4°C for 30 minutes. After this duration the cells were washed three times and resuspended in HBSS before being analysed on the flow cytometer. The percentage of phagocytosis taking place at 4°C was deduced from the phagocytosis percentage at 37°C to remove any background fluorescence or auto-fluorescence.

### Phagocytosis assay of apoptotic neutrophils using confocal microscopy

Neutrophils were isolated from adult healthy controls as previously described [4] and left in 5% CO_2_ at 37°C overnight to undergo apoptosis. Apoptosis was measured using annexin V as previously described [4], in brief neutrophils were removed from culture and re-suspended in HBSS (Invitrogen). Annexin V-FITC (Sigma-Aldrich) was added at a 1/100 dilution and cells were incubated at 4°C for 15 min, cells were pelleted at 400 x g and re-suspended in HBSS before analysis by flow cytometry using a FC500 MPL flow cytometer. Apoptotic neutrophils were stained with pHrodo dye following manufacturer instructions (Life Technologies). Monocytes from adult healthy controls were seeded onto coverslips and differentiated into macrophages as outlined. pHrodo stained apoptotic neutrophils were incubated with the macrophages in the presence of 10% JSLE, JIA or control serum for 2 hrs. Following incubation cells were washed three times in HBSS. To aid orientation, DNA was stained with 1 mg/ml 2-(4-Amidinophenyl)-6-indolecarbamidine dihydrochloride (DAPI) (Sigma-Aldrich), for 5 minutes. Cells were washed with 1xTBS and viewed with a confocal laser-scanning microscope (LeicaDM2500). Images were taken with a 40 x objective and detection settings were kept constant between conditions to compare fluorescence intensities.

### Flow cytometry staining for Mer and ADAM17

Following isolation, cells were washed in HBSS and blocked in mouse serum for 15 minutes at room temperature before being incubated with either Fluorescein-conjugated Mer (R + D), Fluorescein-conjugated ADAM17 (R + D) or isotype control for 30 minutes in the dark at 4°C. Cells were washed and resuspended in HBSS before being immediately analysed on a flow cytometer.

### RNA and RT-PCR analysis

RNA was extracted from CD14^+^ monocytes and macrophage cells using the RNeasy Mini kit (Qiagen Inc., Canada). The concentration and purity of RNA was confirmed by the relative absorbance at 260 nm and measuring the 260/280 nm ratio using a ND-1000 NanoDrop spectrometer (Thermo Scientific). First strand cDNA synthesis was initiated from 100 ng total RNA using random hexamers (Promega, Madison, WI, USA) and avian myeloblastosis virus reverse transcriptase (Promega) using conditions described by the manufacturer in a final volume of 25 μl. The primers used were as follows: β2M forward 5’-TGCCTGCCGTGTGAACCATGT-3’; β2M reverse 5’-TGCGGCATCTTCAAACCTCCATGA-3’; MER forward 5’-CCGCCTGAGCCCGTCAACATT-3’; MER reverse 5’-CTCCGTCAGGCTGGAACAGT-3’. All quantitative real-time PCR took place using the SYBR green fluorescence method with SYBR green qPCR mastermix (Stratagen, La Jolla, CA, USA) as specified by the manufacturer. The real-time PCR reactions took place in triplicate on a MX4000® Multiplex Quantitative QPCR system (Stratagene) using standard thermal cycling conditions. Non-template controls were prepared by replacing the cDNA fraction of the PCR reaction with an equivalent volume of nuclease free water (Promega). Quantification of transcripts took place using the relative standard curve method. mRNA expression for each gene was normalised to the internal standard β2M.

### Measurement of sMer, sTyro3 and sAxl

Soluble receptor concentrations in patient plasma samples were measured using manufactured enzyme-linked immunosorbant assay (ELISA) kits for human total Mer, Tyro3 or Axl (R + D Systems) following manufacturer’s instructions. Samples were diluted 1 in 5 for Mer and Tyro3 and were diluted 1 in 15 for Axl. The ELISA kit was validated using a spike and recovery assay prior to use (mean recovery 113%).

### ADAM17 activity assay

Monocytes were isolated from JSLE, healthy control and JIA patients and were lysed in Cytobuster reagent (Merk Milipore) according to the manufacturer’s instructions at a concentration of 1 × 10^6^ monocytes per 150 μl of reagent. Samples were stored at -20°C until required. ADAM17 activity was analysed in neat samples using InnoZyme™ TACE Activity Kit (Calbiochem) according to the manufacturer’s instructions and the assay was analysed on a fluorescence plate reader.

### Statistical analysis

All data are presented as the mean ± SEM. Comparisons between patients were made using the Mann-Whitney test. All analyses were performed using GraphPad Prism 4 software (Graph Pad Software, San Diego, CA). p < 0.05 were considered significant.

## Results

### Demographic data

Table 1 presents demographic data of the patient groups included in the analysis of the plasma levels of sMer, sTyro3 and sAxl including biomarkers of disease activity and medication at time of sample collection.

**Table 1:** **sMer**, **sTyro3 and sAxl ELISA demographic data**

**Demographics**	**sMer measurement in JSLE patients** **(n** ** = 15)**	**sTyro3 measurement in JSLE patients** **(n** ** = 30)**	**sAxl measurement in JSLE patients** **(n = ** **14)**
Number (%) female	9 (60%)	22 (73%)	11 (79%)
Ethnicity, number			
White British	10	21	10
Asian	5	9	4
Age at sampling, mean (range) years	14.2 (3.5 – 18.7)	14.0 (7.5 - 17.9)	15.5 (10.7-19.4)
Disease duration, mean (range) years	3.0 (0.4 – 9.1)	3.1 (0 – 11.8)	3.5 (0 – 11.5)
Biomarker/disease activity parameter, mean (range)			
ESR, mm/hour (normal 2-8 mm/hour)	15 (1 – 60)	18.5 (<1 – 108)	12.6 (1-50)
CRP, mg/litre (normal 0-8 mg/litre)	11.2 (<4 – 78.9)	6.4 (<4 – 43.3)	4.1 (4-5.6)
C3, gm/litre (normal 1.1 – 1.9)	0.8 (0.5 – 1.4)	1.3 (0.6 – 10.9)	1.1 (0.4 – 1.4)
C4, gm/litre (normal 0.19 – 0.56)	0.14 (0.09 – 0.34)	0.15 (0.08 – 0.46)	0.2 (0.05 – 0.5)
Anti-dsDNA titre, IU/ml (normal <7)	3.1 (14 – 32)	4.9 (0 – 58)	424 (0 – 5408)
C-HAQ score, 0-3, mean (range)	0.27 (0.13 – 2.38)	0.44 (0 – 2.38)	0.64 (0 – 2.25)
Physician’s global assessment of disease activity by VAS, mean (range) mm	19 (5 – 80)	19.4 (0 – 85)	25 (0 – 70)
BILAG-2004			
Number with grade A or grade B	5	11	6
Score, mean (range)	2.6 (1 – 15)	2.9 (0 – 19)	3.8 (0 – 9)
Current medications, number of patients			
Hydroxychloroquine	13	19	10
Methotrexate	0	3	0
Azathioprine	3	4	3
Mycophenolate mofetil	8	13	7
Prednisolone	11	13	6
Prednisolone dosage, mean (range) mg/day	7.4 (1 – 30)	14.4 (3 – 40)	15.8 (2 – 60)
Rituximab	1	0	1
Infliximab	1	2	2

This table presents the demographics, disease and therapeutic profiles, and clinical biomarker results of the JSLE group. Any JSLE patients with a BILAG score of A or B for any organ system at the time of sample collection were considered to have active disease.

### Significantly reduced phagocytosis of E. coli in the presence of JSLE serum

**Figure 1:**
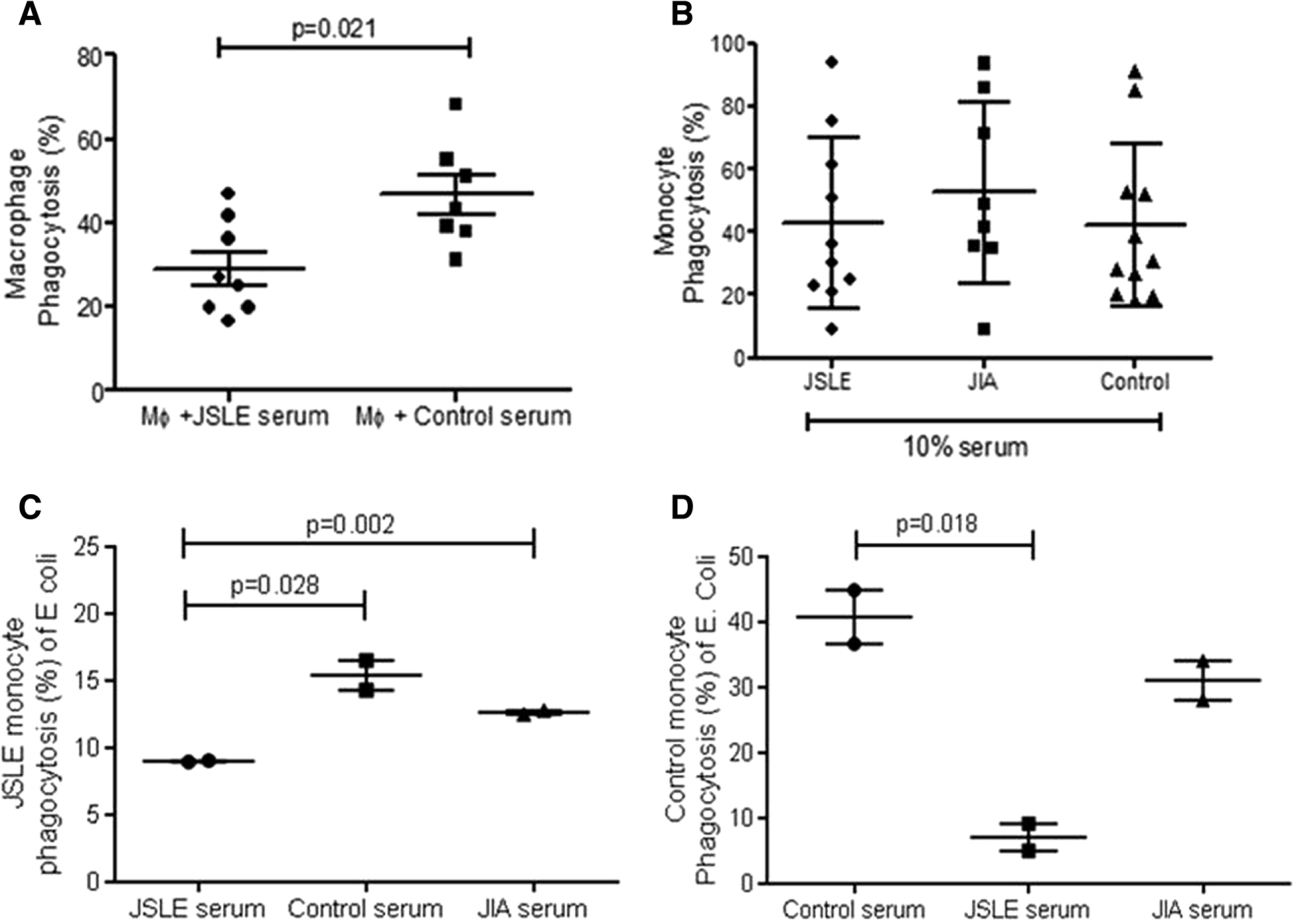
**Phagocytosis level of adult healthy macrophages and monocytes in 10% JSLE, JIA and control serum.** Adult healthy control macrophages were incubated in 10% JSLE (n = 8) or control (n = 7) serum and their ability to phagocytose pHrodo stained E. coli was measured by flow cytometry **(A)**. Macrophages incubated with JSLE serum had significantly decreased phagocytosis ability compared to macrophages incubated with control serum (p = 0.021). Adult healthy monocytes were incubated with 10% JSLE (n = 10), JIA (n = 8) or control (n = 11) serum and their ability to phagocytose pHrodo stained E. coli was measured by flow cytometry. There was no significant difference in the amount of phagocytosis between any of the groups analysed **(B)**. JSLE monocytes were incubated with E coli and either 10% matched JSLE serum, control or JIA serum **(C)**. Levels of E coli phagocytosis were significantly lower when incubated in matched serum but these levels increased in control or JIA serum (p = 0.002 and p = 0.028 respectively). This graph is a representative experiment of the n = 6 experiments performed **(C)**. Control monocytes were incubated with E coli and either 10% matched control serum, JSLE or JIA serum. Incubation of healthy control monocytes with 10% JSLE serum caused a significant decrease in phagocytosis ability in comparison to incubation with control serum (**D**; p = 0.018). This graph is a representative experiment of the n = 5 experiments performed. Data are shown as mean ± SEM and the p values were determined by the Mann Whitney U test.

SLE serum has been shown to contribute to impaired phagocytosis in adult onset SLE [15,16]. In this study, adult healthy control macrophages incubated with 10% JSLE serum (n = 8) had a significantly lower percentage phagocytosis of *E. Coli* measured by flow cytometry compared to macrophages incubated with control serum (n = 7; p = 0.021) (Figure 1A). However there was no significant difference in phagocytosis of *E. Coli* by adult healthy control monocytes incubated in 10% JSLE (n = 11) compared to JIA (n = 8) and healthy control (n = 11) serum (p > 0.05; Figure 1B).

### Control serum can restore phagocytic potential of JSLE monocytes

Monocyte cells isolated from JSLE (n = 5; Figure 1C) or healthy control patients (n = 5; Figure 1D) were incubated with *E coli* in the presence of donor matched serum or JSLE, JIA or healthy control serum. The JSLE monocytes incubated in donor-matched serum had a significantly decreased amount of *E coli* phagocytosis, which could be reversed by incubating the same JSLE monocytes in control (p = 0.0028) or JIA (p = 0.002) serum (Table 2A and Figure 1C). Conversely, healthy control donor monocytes had a higher level of *E coli* phagocytosis which was significantly reduced when the same monocytes cells were incubated in JSLE serum (p = 0.018) (Table 2B and Figure 1D).

**Table 2:** **Phagocytosis of control and JSLE monocytes in 10% JSLE, JIA and control serum**

**(A) Control CD14** ^**+**^ **cells % Phagocytosis**			**(** **B) JSLE CD14** ^**+**^ **cells % Phagocytosis**		
**Control serum**	**JSLE serum**	**JIA serum**	**Control serum**	**JSLE serum**	**JIA serum**
36.7	9.2	34.1	39.5	33.2	21.2
83.6	72.1	81.81	35.8	18.7	62.4
74.72	63.2	69.3	21.6	11.8	20.7
43.73	29.81	42.78	71.1	34.2	64.4
68.3	61.4	76.72	84.81	83.14	93.44
			14.31	9.05	12.8

This table presents the percentage phagocytosis of 5 control and 6 JSLE monocytes represented in Figure 1C and D. Control and JSLE monocytes were incubated with *E coli* and either 10% control serum, JSLE or JIA serum. The percentage of phagocytosis was lower when incubated with JSLE serum which was increased following incubation with control or JIA serum.

### Reduced phagocytosis of apoptotic neutrophils in the presence of JSLE serum compared to controls

**Figure 2:**
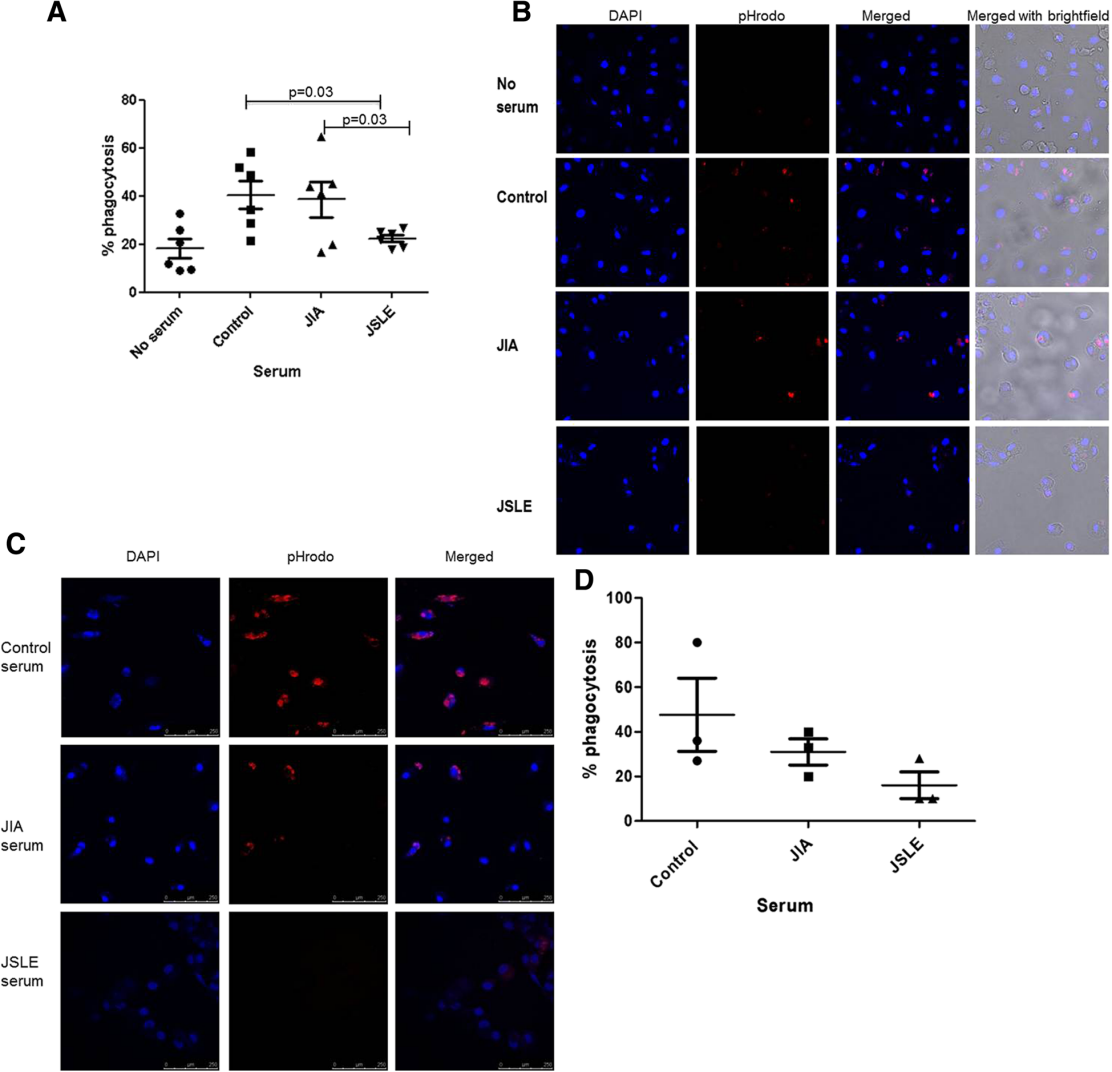
**Decreased phagocytosis of apoptotic neutrophils in the presence of JSLE serum.** Healthy adult control macrophages were incubated with pHrodo stained apoptotic neutrophils in a 10% JSLE, JIA or healthy paediatric control serum environment (n = 6). Macrophages not supplemented with serum were used as a base line control and had the lowest phagocytosis index (phagocytosis index = number of macrophages containing apoptotic material divided by the total number macrophages in the image, averaged across the four areas imaged by confocal microscopy.) Macrophages in JSLE sera had significantly less phagocytic capacity to engulf apoptotic neutrophils compared to those in the control sera group and JIA serum; p = 0.03 **(A)**. Panel **B** shows a series of merged DAPI (blue), pHrodo (red) and Bright field images, from a single representative experiment. Increased red fluorescence in the control and JIA images indicates increased phagocytosis of apoptotic neutrophils compared to JSLE serum. Panel **C** represents a phagocytosis assay using JSLE macrophages incubated with 10% JSLE, JIA or control serum. There is a marked decreased in the amount of red fluorescence in the macrophages incubated with 10% JSLE serum compared to controls indicating less phagocytosis of apoptotic neutrophils by these cells, the % of phagocytosis in each serum is shown in **D**; n = 3.

The effect of JSLE serum on phagocytosis of apoptotic cells was also investigated. Neutrophils that had undergone >60% apoptosis (measured using annexin v staining and flow cytometry) were stained with pHrodo and co- incubated with adult control macrophages with no serum or 10% JSLE, JIA or control serum (n = 7 sera). As expected, those macrophages without a serum enriched environment displayed the lowest level of phagocytosis (Figure 2A). Macrophages incubated with 10% JSLE serum displayed significantly lower phagocytosis of apoptotic neutrophils as measured by confocal microscopy, when compared to macrophages incubated with 10% paediatric control serum (Figure 2A and B). The percentage of phagocytosis following incubation with JIA serum was very similar to levels seen with healthy control serum and significantly higher than with JSLE serum (Figure 2A and B). JSLE monocyte-derived macrophages incubated with 10% JSLE, JIA or control serum resulted in a marked decreased in the amount of phagocytosis of apoptotic neutrophils in the cells incubated with 10% JSLE serum compared to controls (Figure 2C). This again highlights the ability of control serum to potentially restore phagocytic potential of JSLE macrophages.

### Significant reduction of Mer levels on JSLE monocytes

**Figure 3:**
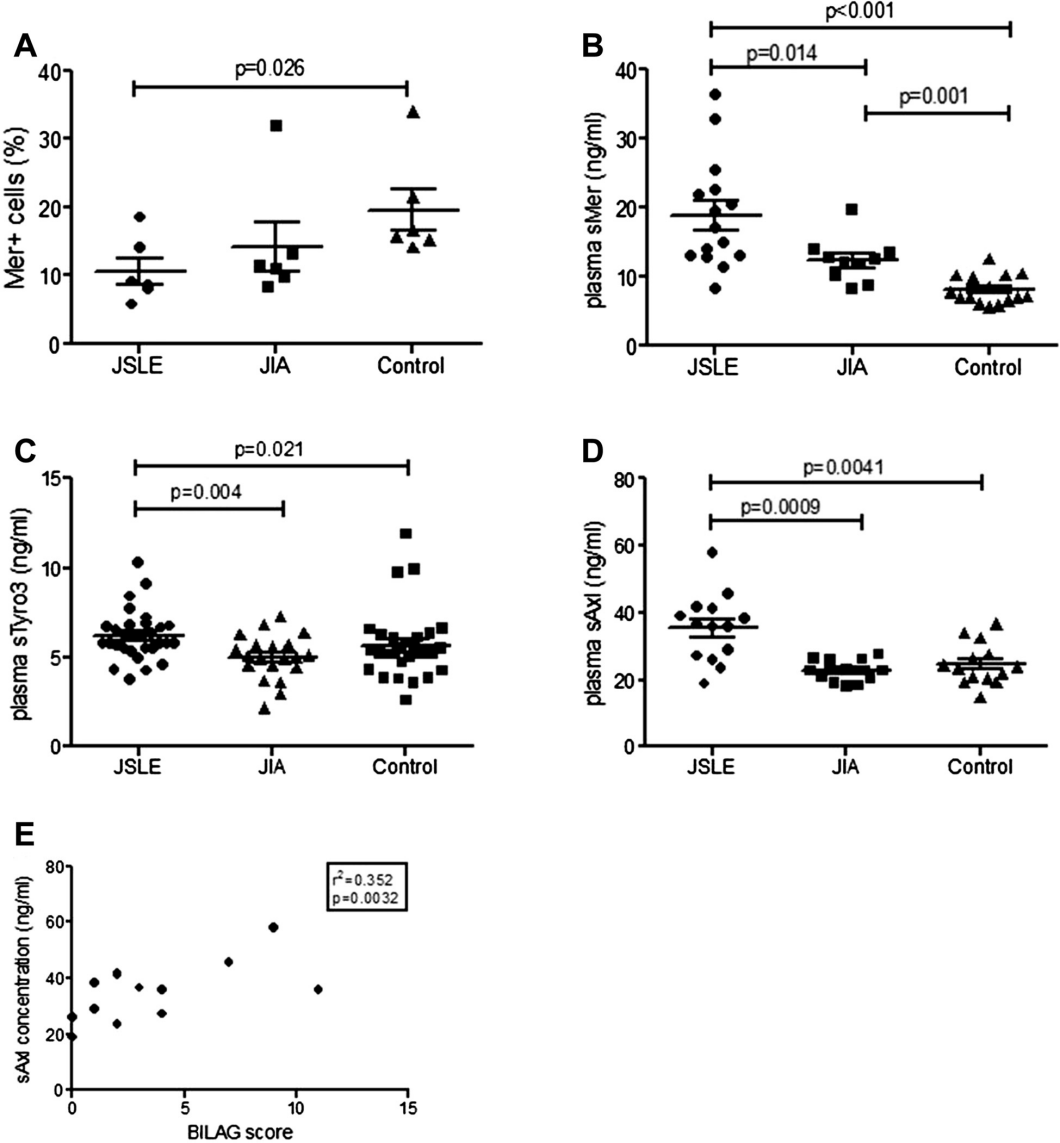
**CD14+ Mer levels and plasma sMer, sTyro and sAXL concentration in JSLE, JIA and control patients.** Levels of cell surface Mer were analysed in JSLE (n = 6), JIA (n = 6) and control (n = 6) patient monocytes **(A)**. Mer was found to be significantly decreased in JSLE monocytes compared to healthy control monocytes (**A**; p = 0.026). JSLE (n = 15), JIA (n = 10) and control (n = 15) patient plasma was analysed by ELISA for sMer concentrations **(B)**. JSLE patients had significantly higher levels of plasma sMer in comparison to JIA and control plasma (**B**; p = 0.014 and p < 0.001 respectively). In addition JIA patients had significantly increased levels of plasma sMer in comparison to healthy controls (B; p = 0.001). JSLE (n = 30), JIA (n = 20) and control (n = 30) patient plasma was analysed by ELISA for sTyro3 concentrations **(C)**. JSLE patients had significantly higher plasma levels of sTyro3 in comparison to JIA and control patients (**C**; p = 0.004 and p = 0.021 respectively). JSLE (n = 14), JIA (n = 12) and control (n = 14) patient plasma was analysed by ELISA for sAxl concentrations **(D)**. JSLE patients had significantly higher plasma levels of sAxl in comparison to JIA and control patients (**D**; p = 0.0009 and p = 0.0041 respectively). BILAG score is significantly correlated with JSLE plasma sAxl concentration (**E**; p = 0.0032). Data are shown as the mean ± SEM and a Mann-Whitney U test was performed to determine significance values.

Monocytes from JSLE (n = 6), JIA (n = 6) and healthy control (n = 6) patients were analysed for Mer receptor levels by flow cytometry (Figure 3A). Mer expression was significantly decreased on JSLE monocytes compared to those of healthy controls (p = 0.026). Levels of Mer were higher on JIA monocytes in comparison to JSLE monocytes but this difference was not statistically significant. There was no significant difference in mRNA expression of Mer in JSLE monocytes (n = 6) compared to controls (n = 6) or between JSLE monocyte-derived macrophages (n = 6) and healthy control macrophages (n = 9) (data not shown).

### sMer, sTyro3 and sAxl concentrations are significantly increased in JSLE plasma

Plasma concentration of sMer was measured in JSLE (n = 15), JIA (n = 10) and healthy control (n = 15) patients (Figure 3B). The concentration of sMer was significantly increased in JSLE plasma compared to both JIA and healthy control (p = 0.014 and p < 0.001 respectively); sMer concentration in JIA patients was significantly increased compared to healthy controls (p = 0.001). JSLE sMer plasma concentration was inversely correlated to patient age (r = -0.714, p = 0.003) but not so for JIA or healthy controls. There was no difference between the sMer concentrations of those on corticosteroid treatment (n = 10; 18.32 ± 2.35) and those not (n = 5; 19.89 ± 4.36; p = 0.95) nor between patients being treated with the immunosuppressants (mycophenolate mofetil, Azathioprine) compared with those who were not (p > 0.05). However, patients receiving hydroxychloroquine (n = 12) showed significantly lower levels of plasma sMer compared to patients not receiving this treatment (n = 3) (p = 0.012) (data not shown). There was no significant correlation between sMer concentration and markers of JSLE disease activity including the British Isles Lupus Assessment Group (BILAG) global score [20]; erythrocyte sedimentation rate (ESR); C reactive protein (CRP); C3 or C4.

JSLE patients (n = 30) had significantly higher plasma levels of sTyro3 in comparison to both JIA (n = 20) and control patients (n = 30;p = 0.004 and p = 0.021 respectively. Figure 3C). These results did not correlate to any markers of disease activity or medication analysed (data not shown).

JSLE patients (n = 14) were found to have significantly higher plasma levels of sAxl in comparison to JIA (n = 12) and control patients (n = 14; p = 0.0009 and p = 0.0041 respectively Figure 3D). JSLE sAxl concentration in plasma was significantly correlated with patient age (r^2^ = 0.53, p = 0.046) and BILAG score (Figure 3E; r^2^ = 0.35, p = 0.032). sAxl levels did not differ significantly with patient’s medication.

### ADAM17 is present within JSLE monocytes

**Figure 4:**
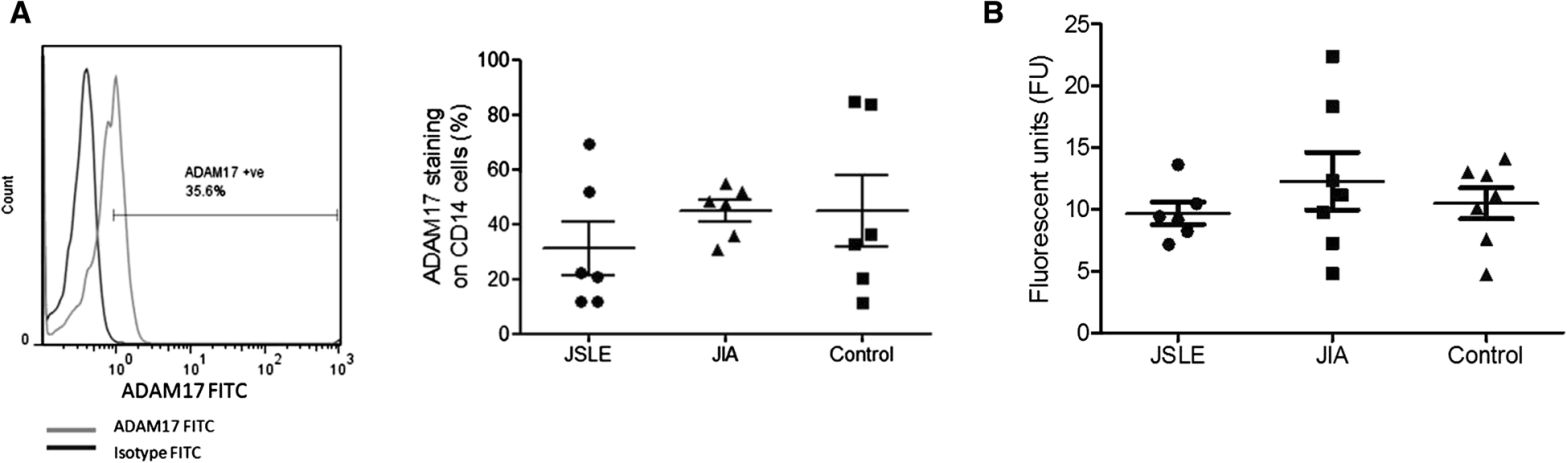
**Protein analysis of ADAM17 enzyme in JSLE, JIA and control monocytes.** Levels of ADAM17 were measured by flow cytometry in JSLE (n = 6), control (n = 6) and JIA (n = 6) CD14 Cells **(A)**. A representative histogram plot of the results is shown **(A)**. CD14 cell lysates were isolated from JSLE (n = 6), JIA (n = 7) and control (n = 7) patients and measured for ADAM17 activity **(B)**. The presence of ADAM17 causes cleavage of a substrate which will fluoresce. The level of fluorescence is measured in fluorescent units (FU).

Due to significantly decreased level of monocyte surface bound Mer and resultant increased levels of sMer within JSLE plasma, the level of an enzyme that can be responsible for Mer cleavage, ADAM17 [12], was investigated. ADAM17 was analysed on the surface of JSLE (n = 6), control (n = 6) and JIA (n = 6) CD14^+^ cells by flow cytometry (Figure 4A). However the level of ADAM17 was not found to be significantly different between the three patient groups. An ADAM17 activity assay was set up to elucidate if the ADAM17 enzyme found within our patient samples was physiologically active and able to cleave its substrate. CD14^+^ cell lysates were isolated from JSLE (n = 6), JIA (n = 7) and control (n = 7) patients and incubated with an ADAM17 substrate which emitted a fluorescent signal upon cleavage by ADAM17. Physiologically active ADAM17 enzyme was present in all samples analysed. However the level of fluorescence was not significantly different between JSLE, JIA and control samples (Figure 4B).

## Discussion

This is the first study to date investigating the TAM receptors and phagocytosis potential of monocytes and macrophages in JSLE and demonstrated reduced phagocytosis, significantly decreased Mer receptor on JSLE monocytes and significantly increased levels of sMer, sAxl and sTyro3 in JSLE plasma.

We have previously identified an increase in neutrophil apoptosis within JSLE patients. Increased apoptotic cell debris has the potential to lead to prolonged exposure of auto-antigens to the immune system and therefore to facilitate the production of antinuclear autoantibodies and the formation of the characteristic immune complexes that cause end organ damage. This study is the first to describe defective phagocytosis in JSLE and shows reduction in phagocytosis of both bacteria and apoptotic cells in the presence of JSLE serum. The importance of serum factors to this fundamental homeostatic process has been highlighted, with a clear reduction in healthy macrophages ability to phagocytise apoptotic neutrophils when incubated with JSLE serum. The dysregulation between cell waste production and removal has been identified as an attractive pathway for therapeutic manipulation [21]. Therefore understanding the mechanisms contributing to this element of JSLE pathogenesis is vitally important.

The Mer receptor is the main receptor used by mouse macrophages to phagocytose apoptotic cells [22]. Mice with a cytoplasmic truncation of Mer, known as *mer*^*kd*^, or lacking the mer receptor (mer^-/-^) have macrophages which are deficient in the clearance of apoptotic cells and also develop progressive lupus-like autoimmunity with antibodies to chromatin, DNA and IgG [23-25]. Mer^-/-^ mice also have apoptotic cell accumulation within germinal centres which results in significantly higher titres of anti-nuclear antibodies (ANA) suggesting that a delayed clearance of apoptotic cells causes a break of peripheral B cell tolerance [25]. Knocking out Mer in mouse macrophages abolishes their ability to phagocytose apoptotic cells; whereas knocking out either Tyro3 or Axl or both receptors reduced macrophage phagocytosis by half [22]. Therefore whilst macrophages may primarily use Mer receptor for the uptake of apoptotic cells, both Tryo3 and Axl also play important roles in macrophage-driven cell clearance. Mer also has an anti-inflammatory role as inhibiting it within a mouse model of LPS-induced acute lung injury enhances inflammatory responses and induces TNFα and IL-1β production [26].

Significantly higher levels of sMer were detected in plasma from JSLE patients compared to both JIA and healthy control patients. This is in keeping with increased levels detected in adult-onset SLE patient plasma compared to healthy controls, correlating with disease activity [27,28]. Increased cleavage of the Mer receptor leading to significantly increased sMer is likely to have a major impact on the phagocytosis rate within JSLE. sMer inhibits phagocytosis in vitro [13] and correlates with disease activity in adult-onset SLE [27,28]. Therefore the significantly increased levels of sMer in JSLE plasma noted here may be a factor causing a significant deficit in apoptotic cell clearance found in adult onset SLE.

ADAM17 is the metalloproteinase enzyme responsible for cleaving Mer [12]. Previous studies have highlighted its importance in several disease conditions especially lung inflammation as prevention of Mer cleavage using the ADAM17 inhibitor TAPI-0 suppressed acute lung injury and inflammation [29,30]. Our study demonstrated all JSLE patient monocytes expressed ADAM17 protein, however there was no significant difference in expression when comparing JSLE to control and JIA monocytes. This data is in-keeping with a previous study demonstrating that ADAM17 was not increased on CD14^+^ isolated from adult SLE patients in comparison to healthy control [31]. The activity assay illustrated that all of the samples analysed contained physiologically active ADAM17 however there was no significant difference in the amount of enzyme activity between JSLE CD14^+^ cells and those from JIA or control patients. Therefore the increase in Mer cleavage leading to significantly increased sMer within JSLE plasma may not be due to increased ADAM17 activity but possibly due to other cleavage inducers in the plasma such as LPS [13] or other currently unknown factors. LPS also inhibits the clearance of neutrophils *in vitro* by suppressing the Mer ligand Gas-6 and by inducing TNFα production which inhibits macrophage phagocytosis in an autocrine manner [32]. Lupus patients are prone to bacterial infections and will often have a flare in disease activity upon infection [33].

As previously mentioned, the other TAM receptors, Tyro3 and Axl also influence cell phagocytosis and both receptors have soluble forms which may act as decoy receptors. JIA plasma sTyro and sAxl levels were observed to be no different to healthy controls. Plasma sTyro3 and sAxl concentrations were however significantly increased in JSLE patients which is consistent with adult data [27,28]. Therefore the effect on phagocytosis of JSLE serum may be reliant on defects in signalling through the combination of all three TAM receptors rather than sMER individually. Mer is also highly expressed on anti-inflammatory M2c macrophages [9] and inhibits the production of the pro-inflammatory cytokine TNFα [34]. Consequently, the significantly higher level of sMer within JIA as well as JSLE plasma may be due to the pro-inflammatory monocytes inactivating Mer by cleavage thereby producing high levels of sMer. Cleavage of Mer could also be causing the JSLE and JIA monocytes to become more activated and increase their secretion of pro-inflammatory molecules such as TNFα which has been shown to be present within the serum of JSLE and JIA patients [35,36].

Analysing the therapeutic regimens of the JSLE patients indicated a significantly lower level of sMer within JSLE patients who were being treated with Hydroxychloroquine (HCQ). Previous studies have demonstrated that the TLR3 ligand poly IC can induce sMer production [12] and SLE-associated autoantigens can act as a TLR3 ligand [37]. HCQ has been shown to inhibit TLR3 function by altering endosomal acidification [38] therefore patients exposed to HCQ may have significantly less plasma sMer due to the drug preventing TLR3 mediated cleavage of Mer. However, as there were only 3 patients in this subgroup we would need to investigate further patients to fully understand the effect of HCQ on sMer. Glucocorticoids increase the mRNA and surface expression of Mer on cultured monocytes and dendritic cells and enhance their phagocytosis of apoptotic neutrophils [9,39,40]. However, we did not find any correlation with the surface level of monocytes Mer expression in patients being treated with the glucocorticoid prednisolone. We also did not find any correlation between the surface level of Mer receptor on monocytes and the level of sMer within the serum of the same patients within JSLE, JIA or controls indicating that the amount of receptor being cleaved is not correlated with the amount of receptor being produced by the monocyte.

This study also demonstrated that healthy macrophages have significantly lower phagocytosis ability to engulf *E. Coli* bacteria in the presence of 10% JSLE serum compared to the same macrophages incubated in 10% control serum. This JSLE serum effect has not previously been demonstrated in the paediatric age group and is in keeping with adult-onset disease [15,16]. We also demonstrated that monocytes from JSLE and healthy control patients also indicated a JSLE serum effect on the phagocytosis. Control monocytes had significantly decreased phagocytosis when exposed to 10% JSLE serum in comparison to control serum. Notably, JSLE neutrophils demonstrate significantly impaired neutrophil phagocytosis of Salmonella-specific LPS compared to those from healthy controls [41]. Major infections are common in JSLE patients and are associated with active disease [42] which our data could suggest may be due to a decreased ability of JSLE phagocytes to engulf and remove organisms such as *E. Coli*.

As already stated we have previously observed increased apoptotic neutrophils in JSLE patients which could be a potential source of auto-antigens. The decreased phagocytosis of apoptotic neutrophils in the presence of JSLE serum compared to controls observed in this study provides further evidence that the production of auto-antibodies to nuclear material found in SLE could be due to increased apoptotic neutrophil apoptosis that aren’t cleared effectively which in part may be due to an increase in cleavage of the TAM receptors.

## Conclusion

In conclusion, this study has demonstrated that phagocytosis is impaired in JSLE. Serum factors appear to play a vital role in inhibiting apoptotic neutrophil clearance and therefore exposing the immune system to autoantigens*.* Although the exact mechanisms are yet to be defined, it is most likely due to a combination of unbalanced elements, which have the potential to become future therapeutic targets.

## Abbreviations

JSLE: Juvenile-onset Systemic Lupus Erythematosus (JSLE)
Tyro 3: Axl
Mer: TAM-receptor
ProS: Protein S
Gas6: growth arrest-specific gene 6
TLR: Toll-like receptor
TNFα: Tumor necrosis factor alpha
IL-6: Interleukin 6
sMer: soluble Mer
ADAM 17: A disintegrin and metalloproteinase protein 17
TACE: Tumour necrosis factor-α-converting enzyme
LPS: Lipopolysaccharide
PMA: Phorbol-12-myristate-13-acetate
ACR: American College of Rheumatology
JIA: Juvenile idiopathic arthritis
ILAR: International League of Arthritis and Rheumatism
PBMC: Peripheral blood mononuclear cells
M-CSF: Macrophage Colony Stimulating Factor
FCS: Fetal calf serum
DAPI: 2-(4-Amidinophenyl)-6-indolecarbamidine dihydrochloride
HBSS: Hanks balanced salt solution
PBS: Phosphate buffered saline
ELISA: Enzyme-linked immunosorbant assay
BILAG: British Isles Lupus Assessment Group
ESR: Erythrocyte sedimentation rate
CRP: C reactive protein
HCQ: Hydroxychloroquine
